# Anæsthetics

**Published:** 1886-06

**Authors:** 


### Anæsthetics.

Dr. R. Harvey Reed thus concludes a paper in the *Fort Wayne Jour. Med. Science* for January:

From this brief review of the anaesthetics most familiar to the profession, from a practical standpoint, we have arrived at the following conclusions:

1. Of all general anaesthetics known, pure sulphuric ether stands at the head for safety, efficiency, and every day practical use.

2. Hydrochlorate of cocaine stands at the head of all known local anaesthetics.

3. Ethidene promises to rival ether, and merits a more general and extended trial.

4. No surgeon should give any anaesthetic without being prepared to resuscitate the patient on the shortest possible notice if necessary; among which preparations nitrite of amyl stands preeminent.

5. No person should be entrusted with the administration of any anaesthetic who is not thoroughly familiar with their physiological action and practical administration.

6. The indiscriminate use of anaesthetics should be strenously guarded against; and especially the practice of leaving such dangerous compounds in the hands of the laity, to be given at liberty whenever they may deem it necessary.

7. The judicious use of anaesthetics under all necessary circumstances should never be omitted; for when properly used by skilled hands they are a glorious haven of peace in the midst of a stormy sea.

